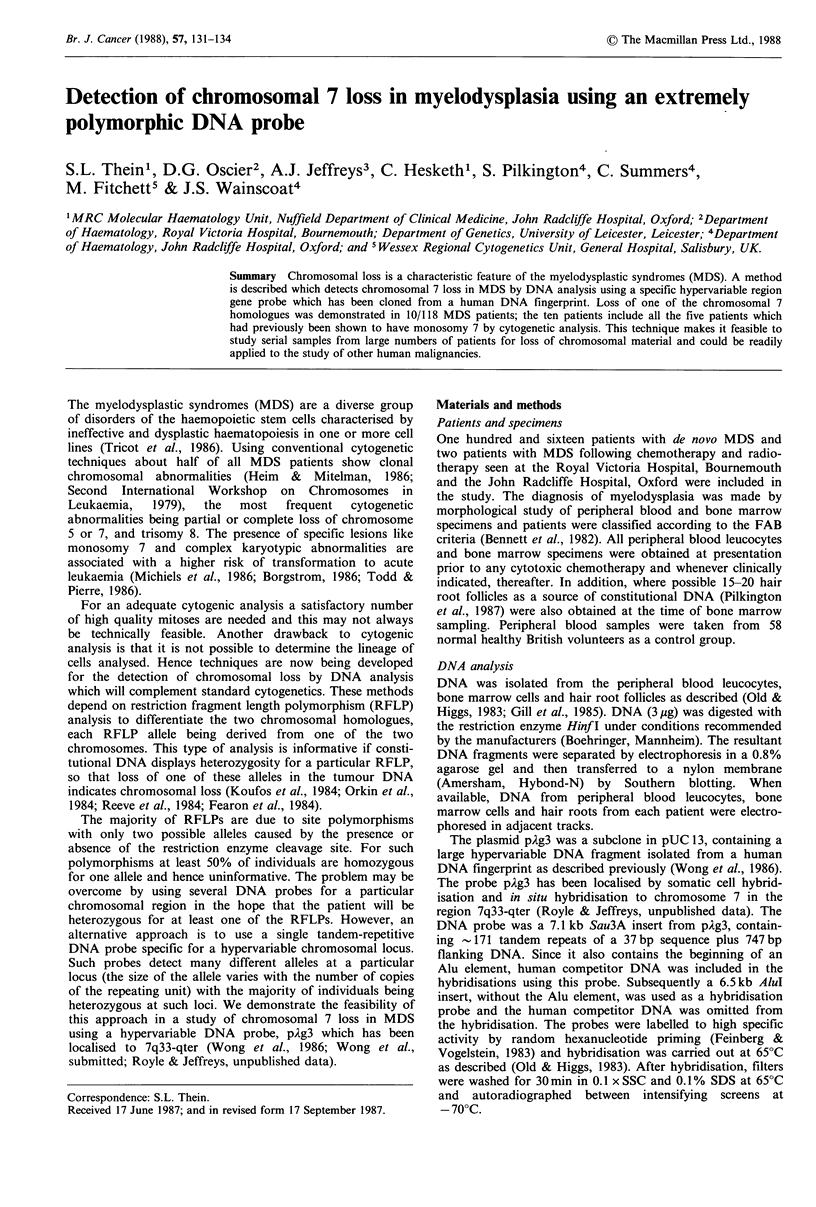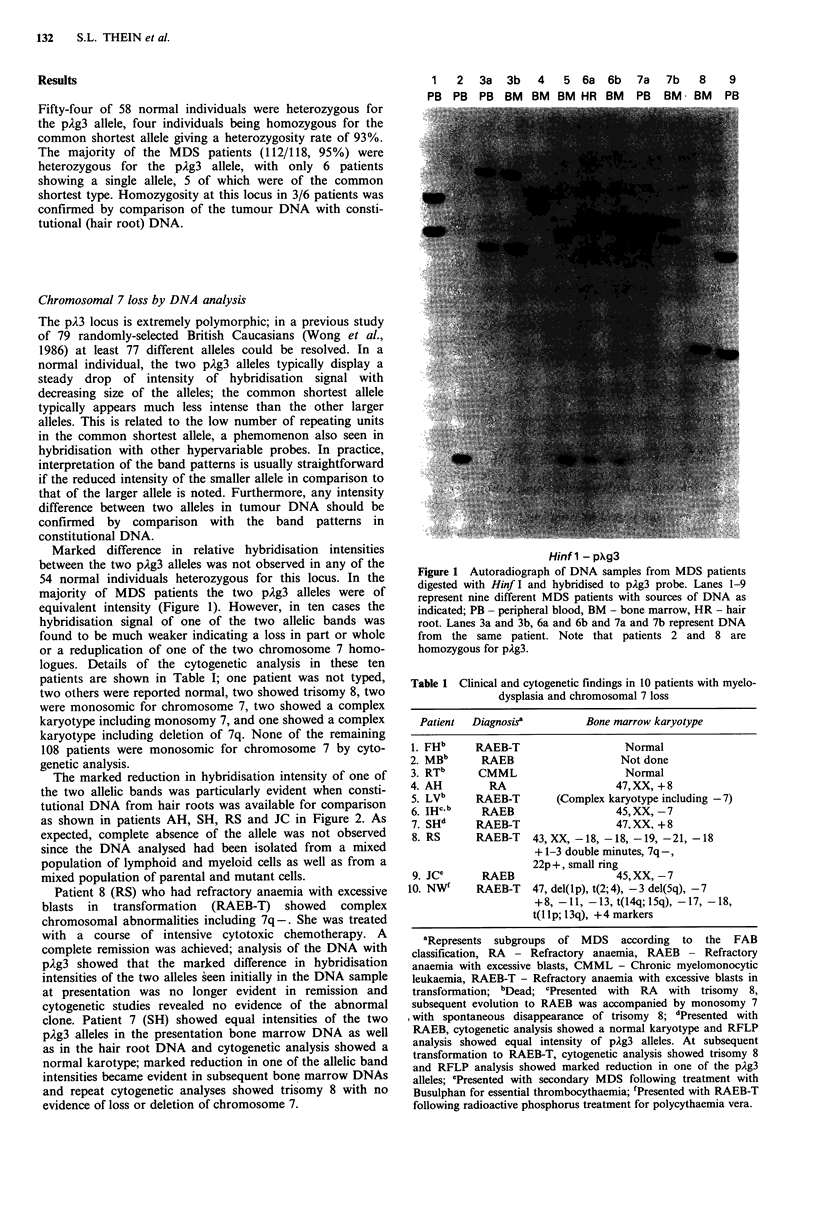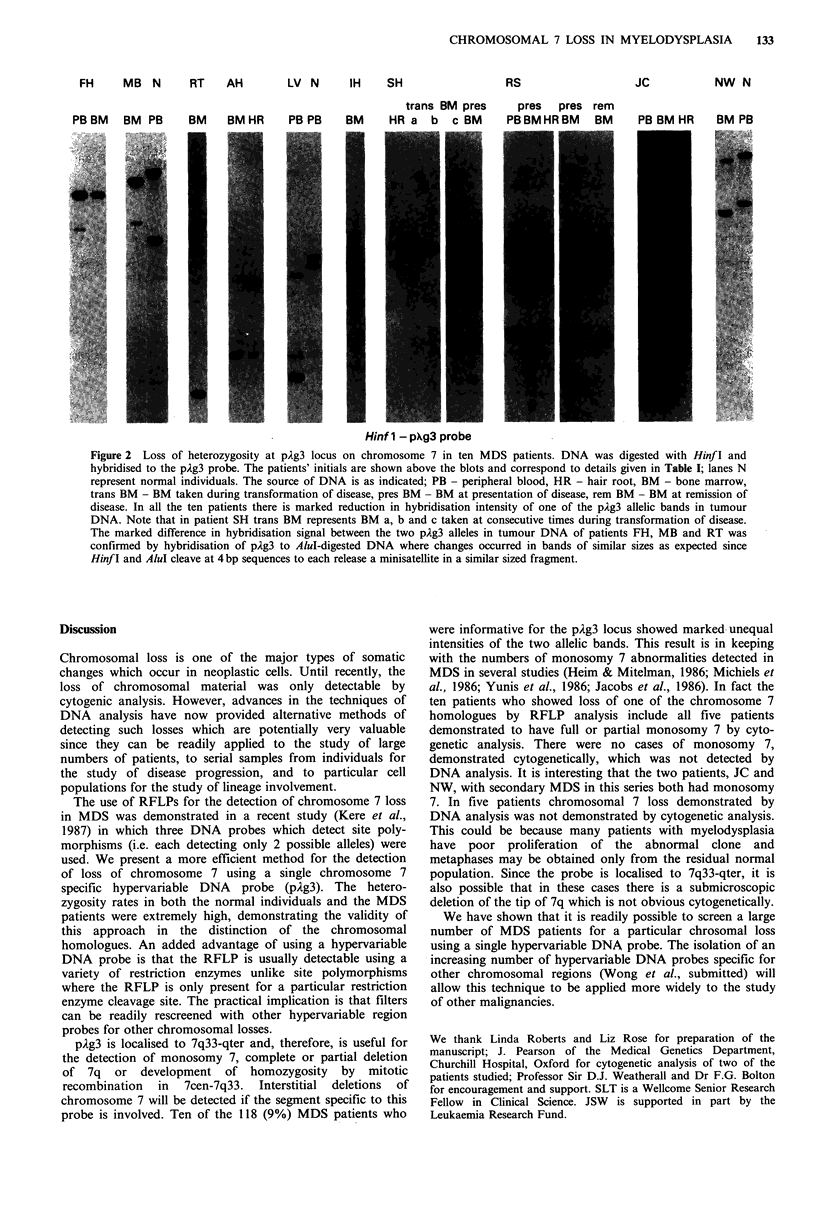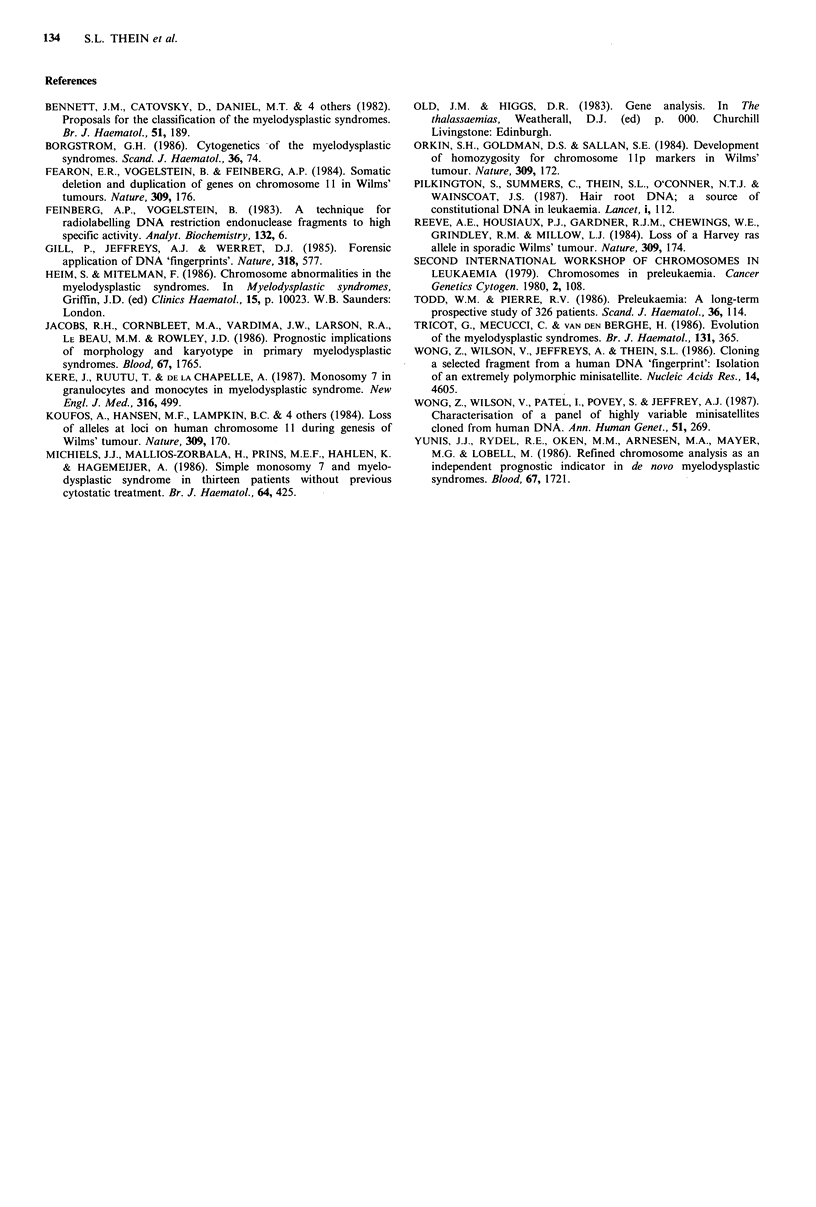# Detection of chromosomal 7 loss in myelodysplasia using an extremely polymorphic DNA probe.

**DOI:** 10.1038/bjc.1988.27

**Published:** 1988-02

**Authors:** S. L. Thein, D. G. Oscier, A. J. Jeffreys, C. Hesketh, S. Pilkington, C. Summers, M. Fitchett, J. S. Wainscoat

**Affiliations:** Nuffield Department of Clinical Medicine, John Radcliffe Hospital, Oxford, UK.

## Abstract

**Images:**


 © The Macmillan Press Ltd., 1988

### Detection of chromosomal 7 loss in myelodysplasia using an extremely polymorphic DNA probe

S.L. Thein[1], D.G. Oscier[2], A.J. Jeffreys[3], C. Hesketh[1], S. Pilkington[4], C. Summers[4], M. Fitchett[5] & J.S. Wainscoat[4]

[1]*MRC Molecular Haematology Unit, Nuffield Department of Clinical Medicine, John Radcliffe Hospital, Oxford;* [2]*Department of Haematology, Royal Victoria Hospital, Bournemouth; Department of Genetics, University of Leicester, Leicester;* [4]*Department of Haematology, John Radcliffe Hospital, Oxford; and* [5]*Wessex Regional Cytogenetics Unit, General Hospital, Salisbury, UK.*

**Summary** Chromosomal loss is a characteristic feature of the myelodysplastic syndromes (MDS). A method is described which detects chromosomal 7 loss in MDS by DNA analysis using a specific hypervariable region gene probe which has been cloned from a human DNA fingerprint. Loss of one of the chromosomal 7 homologues was demonstrated in 10/118 MDS patients; the ten patients include all the five patients which had previously been shown to have monosomy 7 by cytogenetic analysis. This technique makes it feasible to study serial samples from large numbers of patients for loss of chromosomal material and could be readily applied to the study of other human malignancies.

The myelodysplastic syndromes (MDS) are a diverse group of disorders of the haemopoietic stem cells characterised by ineffective and dysplastic haematopoiesis in one or more cell lines (Tricot *et al.*, 1986). Using conventional cytogenetic techniques about half of all MDS patients show clonal chromosomal abnormalities (Heim & Mitelman, 1986; Second International Workshop on Chromosomes in Leukaemia, 1979), the most frequent cytogenetic abnormalities being partial or complete loss of chromosome 5 or 7, and trisomy 8. The presence of specific lesions like monosomy 7 and complex karyotypic abnormalities are associated with a higher risk of transformation to acute leukaemia (Michiels *et al.*, 1986; Borgstrom, 1986; Todd & Pierre, 1986).

For an adequate cytogenic analysis a satisfactory number of high quality mitoses are needed and this may not always be technically feasible. Another drawback to cytogenic analysis is that it is not possible to determine the lineage of cells analysed. Hence techniques are now being developed for the detection of chromosomal loss by DNA analysis which will complement standard cytogenetics. These methods depend on restriction fragment length polymorphism (RFLP) analysis to differentiate the two chromosomal homologues, each RFLP allele being derived from one of the two chromosomes. This type of analysis is informative if constitutional DNA displays heterozygosity for a particular RFLP, so that loss of one of these alleles in the tumour DNA indicates chromosomal loss (Koufos *et al.*, 1984; Orkin *et al.*, 1984; Reeve *et al.*, 1984; Fearon *et al.*, 1984).

The majority of RFLPs are due to site polymorphisms with only two possible alleles caused by the presence or absence of the restriction enzyme cleavage site. For such polymorphisms at least 50% of individuals are homozygous for one allele and hence uninformative. The problem may be overcome by using several DNA probes for a particular chromosomal region in the hope that the patient will be heterozygous for at least one of the RFLPs. However, an alternative approach is to use a single tandem-repetitive DNA probe specific for a hypervariable chromosomal locus. Such probes detect many different alleles at a particular locus (the size of the allele varies with the number of copies of the repeating unit) with the majority of individuals being heterozygous at such loci. We demonstrate the feasibility of this approach in a study of chromosomal 7 loss in MDS using a hypervariable DNA probe, pλg3 which has been localised to 7q33-qter (Wong *et al.*, 1986; Wong *et al.*, submitted; Royle & Jeffreys, unpublished data).

Correspondence: S.L. Thein.
Received 17 June 1987; and in revised form 17 September 1987.

### Materials and methods

### *Patients and specimens*

One hundred and sixteen patients with *de novo* MDS and two patients with MDS following chemotherapy and radiotherapy seen at the Royal Victoria Hospital, Bournemouth and the John Radcliffe Hospital, Oxford were included in the study. The diagnosis of myelodysplasia was made by morphological study of peripheral blood and bone marrow specimens and patients were classified according to the FAB criteria (Bennett *et al.*, 1982). All peripheral blood leucocytes and bone marrow specimens were obtained at presentation prior to any cytotoxic chemotherapy and whenever clinically indicated, thereafter. In addition, where possible 15–20 hair root follicles as a source of constitutional DNA (Pilkington *et al.*, 1987) were also obtained at the time of bone marrow sampling. Peripheral blood samples were taken from 58 normal healthy British volunteers as a control group.

### *DNA analysis*

DNA was isolated from the peripheral blood leucocytes, bone marrow cells and hair root follicles as described (Old & Higgs, 1983; Gill *et al.*, 1985). DNA (3 μg) was digested with the restriction enzyme *Hinf*I under conditions recommended by the manufacturers (Boehringer, Mannheim). The resultant DNA fragments were separated by electrophoresis in a 0.8% agarose gel and then transferred to a nylon membrane (Amersham, Hybond-N) by Southern blotting. When available, DNA from peripheral blood leucocytes, bone marrow cells and hair roots from each patient were electrophoresed in adjacent tracks.

The plasmid pλg3 was a subclone in pUC13, containing a large hypervariable DNA fragment isolated from a human DNA fingerprint as described previously (Wong *et al.*, 1986). The probe pλg3 has been localised by somatic cell hybridisation and *in situ* hybridisation to chromosome 7 in the region 7q33-qter (Royle & Jeffreys, unpublished data). The DNA probe was a 7.1 kb *Sau*3A insert from pλg3, containing ~171 tandem repeats of a 37 bp sequence plus 747 bp flanking DNA. Since it also contains the beginning of an Alu element, human competitor DNA was included in the hybridisations using this probe. Subsequently a 6.5 kb *Alu*I insert, without the Alu element, was used as a hybridisation probe and the human competitor DNA was omitted from the hybridisation. The probes were labelled to high specific activity by random hexanucleotide priming (Feinberg & Vogelstein, 1983) and hybridisation was carried out at 65°C as described (Old & Higgs, 1983). After hybridisation, filters were washed for 30 min in 0.1 × SSC and 0.1% SDS at 65°C and autoradiographed between intensifying screens at −70°C.

### Results

Fifty-four of 58 normal individuals were heterozygous for the pλg3 allele, four individuals being homozygous for the common shortest allele giving a heterozygosity rate of 93%. The majority of the MDS patients (112/118, 95%) were heterozygous for the pλg3 allele, with only 6 patients showing a single allele, 5 of which were of the common shortest type. Homozygosity at this locus in 3/6 patients was confirmed by comparison of the tumour DNA with constitutional (hair root) DNA.

### *Chromosomal 7 loss by DNA analysis*

The pλ3 locus is extremely polymorphic; in a previous study of 79 randomly-selected British Caucasians (Wong *et al.*, 1986) at least 77 different alleles could be resolved. In a normal individual, the two pλg3 alleles typically display a steady drop of intensity of hybridisation signal with decreasing size of the alleles; the common shortest allele typically appears much less intense than the other larger alleles. This is related to the low number of repeating units in the common shortest allele, a phemomenon also seen in hybridisation with other hypervariable probes. In practice, interpretation of the band patterns is usually straightforward if the reduced intensity of the smaller allele in comparison to that of the larger allele is noted. Furthermore, any intensity difference between two alleles in tumour DNA should be confirmed by comparison with the band patterns in constitutional DNA.

Marked difference in relative hybridisation intensities between the two pλg3 alleles was not observed in any of the 54 normal individuals heterozygous for this locus. In the majority of MDS patients the two pλg3 alleles were of equivalent intensity (Figure 1). However, in ten cases the hybridisation signal of one of the two allelic bands was found to be much weaker indicating a loss in part or whole or a reduplication of one of the two chromosome 7 homologues. Details of the cytogenetic analysis in these ten patients are shown in Table I; one patient was not typed, two others were reported normal, two showed trisomy 8, two were monosomic for chromosome 7, two showed a complex karyotype including monosomy 7, and one showed a complex karyotype including deletion of 7q. None of the remaining 108 patients were monosomic for chromosome 7 by cytogenetic analysis.

The marked reduction in hybridisation intensity of one of the two allelic bands was particularly evident when constitutional DNA from hair roots was available for comparison as shown in patients AH, SH, RS and JC in Figure 2. As expected, complete absence of the allele was not observed since the DNA analysed had been isolated from a mixed population of lymphoid and myeloid cells as well as from a mixed population of parental and mutant cells.

Patient 8 (RS) who had refractory anaemia with excessive blasts in transformation (RAEB-T) showed complex chromosomal abnormalities including 7q−. She was treated with a course of intensive cytotoxic chemotherapy. A complete remission was achieved; analysis of the DNA with pλg3 showed that the marked difference in hybridisation intensities of the two alleles seen initially in the DNA sample at presentation was no longer evident in remission and cytogenetic studies revealed no evidence of the abnormal clone. Patient 7 (SH) showed equal intensities of the two pλg3 alleles in the presentation bone marrow DNA as well as in the hair root DNA and cytogenetic analysis showed a normal karotype; marked reduction in one of the allelic band intensities became evident in subsequent bone marrow DNAs and repeat cytogenetic analyses showed trisomy 8 with no evidence of loss or deletion of chromosome 7.

**Figure 1** Autoradiograph of DNA samples from MDS patients digested with *Hinf* I and hybridised to pλg3 probe. Lanes 1–9 represent nine different MDS patients with sources of DNA as indicated; PB – peripheral blood, BM – bone marrow, HR – hair root. Lanes 3a and 3b, 6a and 6b and 7a and 7b represent DNA from the same patient. Note that patients 2 and 8 are homozygous for pλg3.

**Table 1** Clinical and cytogenetic findings in 10 patients with myelodysplasia and chromosomal 7 loss

| *Patient* | *Diagnosis*[a] | *Bone marrow karyotype* |
|---|---|---|
| 1. FH[b] | RAEB-T | Normal |
| 2. MB[b] | RAEB | Not done |
| 3. RT[b] | CMML | Normal |
| 4. AH | RA | 47,XX, +8 |
| 5. LV[b] | RAEB-T | (Complex karyotype including −7) |
| 6. IH[c,b] | RAEB | 45,XX, −7 |
| 7. SH[d] | RAEB-T | 47,XX, +8 |
| 8. RS | RAEB-T | 43, XX, −18, −18, −19, −21, −18 +1-3 double minutes, 7q−, 22p+, small ring |
| 9. JC[e] | RAEB | 45,XX, −7 |
| 10. NW[f] | RAEB-T | 47, del(1p), t(2;4), −3 del(5q), −7 +8, −11, −13, t(14q;15q), −17, −18, t(11p;13q), +4 markers |

[a]Represents subgroups of MDS according to the FAB classification, RA – Refractory anaemia, RAEB – Refractory anaemia with excessive blasts, CMML – Chronic myelomonocytic leukaemia, RAEB-T – Refractory anaemia with excessive blasts in transformation; [b]Dead; [c]Presented with RA with trisomy 8, subsequent evolution to RAEB was accompanied by monosomy 7 with spontaneous disappearance of trisomy 8; [d]Presented with RAEB, cytogenetic analysis showed a normal karyotype and RFLP analysis showed equal intensity of pλg3 alleles. At subsequent transformation to RAEB-T, cytogenetic analysis showed trisomy 8 and RFLP analysis showed marked reduction in one of the pλg3 alleles; [e]Presented with secondary MDS following treatment with Busulphan for essential thrombocythaemia; [f]Presented with RAEB-T following radioactive phosphorus treatment for polycythaemia vera.

**Figure 2** Loss of heterozygosity at pλg3 locus on chromosome 7 in ten MDS patients. DNA was digested with *Hinf*I and hybridised to the pλg3 probe. The patients' initials are shown above the blots and correspond to details given in **Table I**; lanes N represent normal individuals. The source of DNA is as indicated; PB – peripheral blood, HR – hair root, BM – bone marrow, trans BM – BM taken during transformation of disease, pres BM – BM at presentation of disease, rem BM – BM at remission of disease. In all the ten patients there is marked reduction in hybridisation intensity of one of the pλg3 allelic bands in tumour DNA. Note that in patient SH trans BM represents BM a, b and c taken at consecutive times during transformation of disease. The marked difference in hybridisation signal between the two pλg3 alleles in tumour DNA of patients FH, MB and RT was confirmed by hybridisation of pλg3 to *Alu*I-digested DNA where changes occurred in bands of similar sizes as expected since *Hinf*I and *Alu*I cleave at 4 bp sequences to each release a minisatellite in a similar sized fragment.

### Discussion

Chromosomal loss is one of the major types of somatic changes which occur in neoplastic cells. Until recently, the loss of chromosomal material was only detectable by cytogenic analysis. However, advances in the techniques of DNA analysis have now provided alternative methods of detecting such losses which are potentially very valuable since they can be readily applied to the study of large numbers of patients, to serial samples from individuals for the study of disease progression, and to particular cell populations for the study of lineage involvement.

The use of RFLPs for the detection of chromosome 7 loss in MDS was demonstrated in a recent study (Kere *et al.*, 1987) in which three DNA probes which detect site polymorphisms (i.e. each detecting only 2 possible alleles) were used. We present a more efficient method for the detection of loss of chromosome 7 using a single chromosome 7 specific hypervariable DNA probe (pλg3). The heterozygosity rates in both the normal individuals and the MDS patients were extremely high, demonstrating the validity of this approach in the distinction of the chromosomal homologues. An added advantage of using a hypervariable DNA probe is that the RFLP is usually detectable using a variety of restriction enzymes unlike site polymorphisms where the RFLP is only present for a particular restriction enzyme cleavage site. The practical implication is that filters can be readily rescreened with other hypervariable region probes for other chromosomal losses.

pλg3 is localised to 7q33-qter and, therefore, is useful for the detection of monosomy 7, complete or partial deletion of 7q or development of homozygosity by mitotic recombination in 7cen-7q33. Interstitial deletions of chromosome 7 will be detected if the segment specific to this probe is involved. Ten of the 118 (9%) MDS patients who were informative for the pλg3 locus showed marked unequal intensities of the two allelic bands. This result is in keeping with the numbers of monosomy 7 abnormalities detected in MDS in several studies (Heim & Mitelman, 1986; Michiels *et al.*, 1986; Yunis *et al.*, 1986; Jacobs *et al.*, 1986). In fact the ten patients who showed loss of one of the chromosome 7 homologues by RFLP analysis include all five patients demonstrated to have full or partial monosomy 7 by cytogenetic analysis. There were no cases of monosomy 7, demonstrated cytogenetically, which was not detected by DNA analysis. It is interesting that the two patients, JC and NW, with secondary MDS in this series both had monosomy 7. In five patients chromosomal 7 loss demonstrated by DNA analysis was not demonstrated by cytogenetic analysis. This could be because many patients with myelodysplasia have poor proliferation of the abnormal clone and metaphases may be obtained only from the residual normal population. Since the probe is localised to 7q33-qter, it is also possible that in these cases there is a submicroscopic deletion of the tip of 7q which is not obvious cytogenetically.

We have shown that it is readily possible to screen a large number of MDS patients for a particular chrosomal loss using a single hypervariable DNA probe. The isolation of an increasing number of hypervariable DNA probes specific for other chromosomal regions (Wong *et al.*, submitted) will allow this technique to be applied more widely to the study of other malignancies.

We thank Linda Roberts and Liz Rose for preparation of the manuscript; J. Pearson of the Medical Genetics Department, Churchill Hospital, Oxford for cytogenetic analysis of two of the patients studied; Professor Sir D.J. Weatherall and Dr F.G. Bolton for encouragement and support. SLT is a Wellcome Senior Research Fellow in Clinical Science. JSW is supported in part by the Leukaemia Research Fund.

### References

BENNETT, J.M., CATOVSKY, D., DANIEL, M.T. & 4 others (1982). Proposals for the classification of the myelodysplastic syndromes. *Br. J. Haematol.*, **51**, 189.

BORGSTROM, G.H. (1986). Cytogenetics of the myelodysplastic syndromes. *Scand. J. Haematol.*, **36**, 74.

FEARON, E.R., VOGELSTEIN, B. & FEINBERG, A.P. (1984). Somatic deletion and duplication of genes on chromosome 11 in Wilms' tumours. *Nature*, **309**, 176.

FEINBERG, A.P., VOGELSTEIN, B. (1983). A technique for radiolabelling DNA restriction endonuclease fragments to high specific activity. *Analyt. Biochemistry*, **132**, 6.

GILL, P., JEFFREYS, A.J. & WERRET, D.J. (1985). Forensic application of DNA 'fingerprints'. *Nature*, **318**, 577.

HEIM, S. & MITELMAN, F. (1986). Chromosome abnormalities in the myelodysplastic syndromes. In *Myelodysplastic syndromes*, Griffin, J.D. (ed) *Clinics Haematol.*, **15**, p. 10023. W.B. Saunders: London.

JACOBS, R.H., CORNBLEET, M.A., VARDIMA, J.W., LARSON, R.A., LE BEAU, M.M. & ROWLEY, J.D. (1986). Prognostic implications of morphology and karyotype in primary myelodysplastic syndromes. *Blood*, **67**, 1765.

KERE, J., RUUTU, T. & DE LA CHAPELLE, A. (1987). Monosomy 7 in granulocytes and monocytes in myelodysplastic syndrome. *New Engl. J. Med.*, **316**, 499.

KOUFOS, A., HANSEN, M.F., LAMPKIN, B.C. & 4 others (1984). Loss of alleles at loci on human chromosome 11 during genesis of Wilms' tumour. *Nature*, **309**, 170.

MICHIELS, J.J., MALLIOS-ZORBALA, H., PRINS, M.E.F., HAHLEN, K. & HAGEMEIJER, A. (1986). Simple monosomy 7 and myelodysplastic syndrome in thirteen patients without previous cytostatic treatment. *Br. J. Haematol.*, **64**, 425.

OLD, J.M. & HIGGS, D.R. (1983). Gene analysis. In *The thalassaemias*, Weatherall, D.J. (ed) p. 000. Churchill Livingstone: Edinburgh.

ORKIN, S.H., GOLDMAN, D.S. & SALLAN, S.E. (1984). Development of homozygosity for chromosome 11p markers in Wilms' tumour. *Nature*, **309**, 172.

PILKINGTON, S., SUMMERS, C., THEIN, S.L., O'CONNER, N.T.J. & WAINSCOAT, J.S. (1987). Hair root DNA; a source of constitutional DNA in leukaemia. *Lancet*, **i**, 112.

REEVE, A.E., HOUSIAUX, P.J., GARDNER, R.J.M., CHEWINGS, W.E., GRINDLEY, R.M. & MILLOW, L.J. (1984). Loss of a Harvey ras allele in sporadic Wilms' tumour. *Nature*, **309**, 174.

SECOND INTERNATIONAL WORKSHOP OF CHROMOSOMES IN LEUKAEMIA (1979). Chromosomes in preleukaemia. *Cancer Genetics Cytogen.* 1980, **2**, 108.

TODD, W.M. & PIERRE, R.V. (1986). Preleukaemia: A long-term prospective study of 326 patients. *Scand. J. Haematol.*, **36**, 114.

TRICOT, G., MECUCCI, C. & VAN DEN BERGHE, H. (1986). Evolution of the myelodysplastic syndromes. *Br. J. Haematol.*, **131**, 365.

WONG, Z., WILSON, V., JEFFREYS, A. & THEIN, S.L. (1986). Cloning a selected fragment from a human DNA 'fingerprint': Isolation of an extremely polymorphic minisatellite. *Nucleic Acids Res.*, **14**, 4605.

WONG, Z., WILSON, V., PATEL, I., POVEY, S. & JEFFREY, A.J. (1987). Characterisation of a panel of highly variable minisatellites cloned from human DNA. *Ann. Human Genet.*, **51**, 269.

YUNIS, J.J., RYDEL, R.E., OKEN, M.M., ARNESEN, M.A., MAYER, M.G. & LOBELL, M. (1986). Refined chromosome analysis as an independent prognostic indicator in *de novo* myelodysplastic syndromes. *Blood*, **67**, 1721.